# The use of the hypervariable P8 region of *trn*L(UAA) intron for identification of orchid species: Evidence from restriction site polymorphism analysis

**DOI:** 10.1371/journal.pone.0196680

**Published:** 2018-05-02

**Authors:** Rajkumar Kishor, G. J. Sharma

**Affiliations:** Department of Life Sciences, Manipur University, Imphal, Manipur, India; University of Naples Federico II, ITALY

## Abstract

The P8 stem-loop region of the *trn*L intron, which is known to be hypervariable in size with multiple repeat motifs and created difficulties in alignment, is always excluded in phylogenetic as well as barcode analyses. This region was investigated for species discrimination in 98 taxa of orchids belonging to the tribe Vandeae using in silico mapping of restriction site polymorphism. The length of the P8 regions varied from 200 nucleotides in *Aerides rosea* to 669 nucleotides in *Dendrophylax sallei*. Forty two taxa had unique lengths, while as many as eight shared a common length of 521 nucleotides. Of the 35 restriction endonucleases producing digestions in the P8 regions, three, viz., *Ags*I, *Apo*I and *Tsp*DTI turned out to have recognition sites across all the 98 taxa being studied. When their restriction data were combined, 92 taxa could be discriminated leaving three taxon pairs. However, *Acampe papillosa* and *Aeranthes arachnites* despite having similar restriction sites differed in their P8 lengths. This is the first report on thorough investigation of the P8 region of *trn*L intron for search of species specific restriction sites and hence its use as a potential plant DNA barcode.

## Introduction

For the past few decades there has been a hunt for a short DNA segment which can be used as a universal marker, popularly termed as *DNA Barcode*, for identification of faunal and floral species inhabiting this planet. For animal identification the mitochondrial gene *cytochrome c oxidase subunit 1* (*COX*1) has proved successful [1] however, there is still difficulty in fixing a universal barcode for plants because of a more complex genetic background. Various nuclear and plastid coding and non-coding loci, *viz*., ITS1, ITS2, *acc*D, *mat*K, *ndh*J, *rpo*B, *rpo*C1, *ycf*5, *atp*F-H, *psb*K-I, *rbc*L, *rbc*La, *trn*H-*psb*A, *trn*L-F, etc. have been tested for validation either singly or in combination of two or more loci [2–4].

The chloroplast *trn*L-F non-coding region includes the *trn*L(UAA) intron ranging from 350 to 600 base pairs (bp) and the intergeneric spacer between *trn*L(UAA) 3′ exon and the *trn*F(GAA) gene [5,6]. The *trn*L(UAA) intron interrupts the anticodon loop of the tRNA_Leu,_ which is encoded in the large single copy region of the plastid genome_._ In the chloroplast DNA, *trn*L is the only Group I intron region having conserved secondary structure [7,8] with alternation of conserved and variable regions [9]. They are capable of catalyzing their own splicing from the flanking exons. The secondary structure of *trn*L intron contains regions of complimentary sequences that form nine stem-loop structures (P1-P9) [10]. Within these stems there are four regions (P, Q, R and S) conserved in primary sequences among all group I introns [11] and they are known as the catalytic core [12]. The P8 stem-loop region of the *trn*L intron is known to be hypervariable in size with multiple repeat motifs [12,13] and created difficulties in alignment. Therefore, this region is always excluded in phylogenetic as well as barcode analyses [14–17].

Orchidaceae comprises of 850 genera and 20,000 species which are arranged in five subfamilies, 22 tribes and 70 sub-tribes [18]. The tribe Vandeae consists of five sub-tribes, 139 genera with 2600 species of monopodial epiphytes distributed in tropical America, tropical and southern Africa, tropical and sub-tropical Asia, eastern Australia and Tasmania, much of the tropical Pacific south to New Zealand and east to Tahiti [19]. Considering the hypervariability to be an intrinsic property of the P8 region of *trn*L(UAA) intron, the present investigation was undertaken to find out whether the locus had any potential in discrimination of closely related species belonging to the tribe Vandeae based on in silico restriction site polymorphism analysis.

## Materials and methods

### Gene sequences

One hundred and twenty five sequences of tRNA_Leu_ (*trn*L) gene for orchids belonging to tribe Vandeae were downloaded from GenBank. These included ten sequences (GU185926, GU185928, GU185931, GU185932, GU185933, GU185934, GU185935, GU185938, GU185939 and GU185940) generated and submitted by the first author. Methods followed for DNA extraction and PCR amplification were given by Kishor and Sunitibala [20]. Sequencing was done using 3730 DNA Analyzer (Applied Biosystems, Warrington, UK) available at DNA Sequencing Facility, University of Delhi, South Campus. The primer pair ‘c’ (3′CGAAATCGGTAGACGCTACG5′) and ‘d’ (3′GGGGATAGAGGGACTTGAAC5′) [5] were used for amplification as well as sequencing.

### In silico analysis of restriction site polymorphism for species discrimination

The *trn*L intron borders were delimited by identifying the binding sites of the primers ‘c’ and ‘d’. We looked for the P8 region by delimiting the borders following the method of Borsch et al. [13] and by considering the secondary structures of *trn*L of *Campylopus flexuous* [21] and *Nymphaea odorata* [13]. The correctness of the borders was again verified by considering the secondary structures of the P8 regions drawn for *Aerides odorata*, *A*. *sukauensis* and *A*. *krabiensis* [16].

In silico restriction mapping was done using the online software *RestrictionMapper* Version 3 (http://restrictionmapper.org/). Selection was made to include all commercially available restriction enzymes and find the base pair position where each enzyme cut the *trn*L P8 region for all the taxa. The program was set to allow providing maximum cuts with a minimum site length of 5 nucleotides. As many as 35 restriction endonucleases were found to have recognition sites in the P8 regions; however, only four, viz., *Ags*I, *Apo*I, *Tsp*DTI and *Vsp*I (five and six base cutters) were selected for the analysis because each of them had their recognition sites in the region across all the taxa.

The in silico mapped restrictions produced by the four enzymes were scored by presence (1) or absence (0). Further analyses were done using the computer package NTSYS [22]. Similarity matrix was generated using the program Qualitative in NTSYS 2.20e package. This matrix was subjected to the unweighted pair group method with arithmetical averages (UPGMA). Cluster analysis was performed on the similarity matrix with the SAHN program using UPGMA and the dendrograms were generated with the TREE program. Analysis was done for each enzyme as well as for combination of two, three and four of them.

## Results

### Length variation of *trn*L P8 region

**Fig 1:**
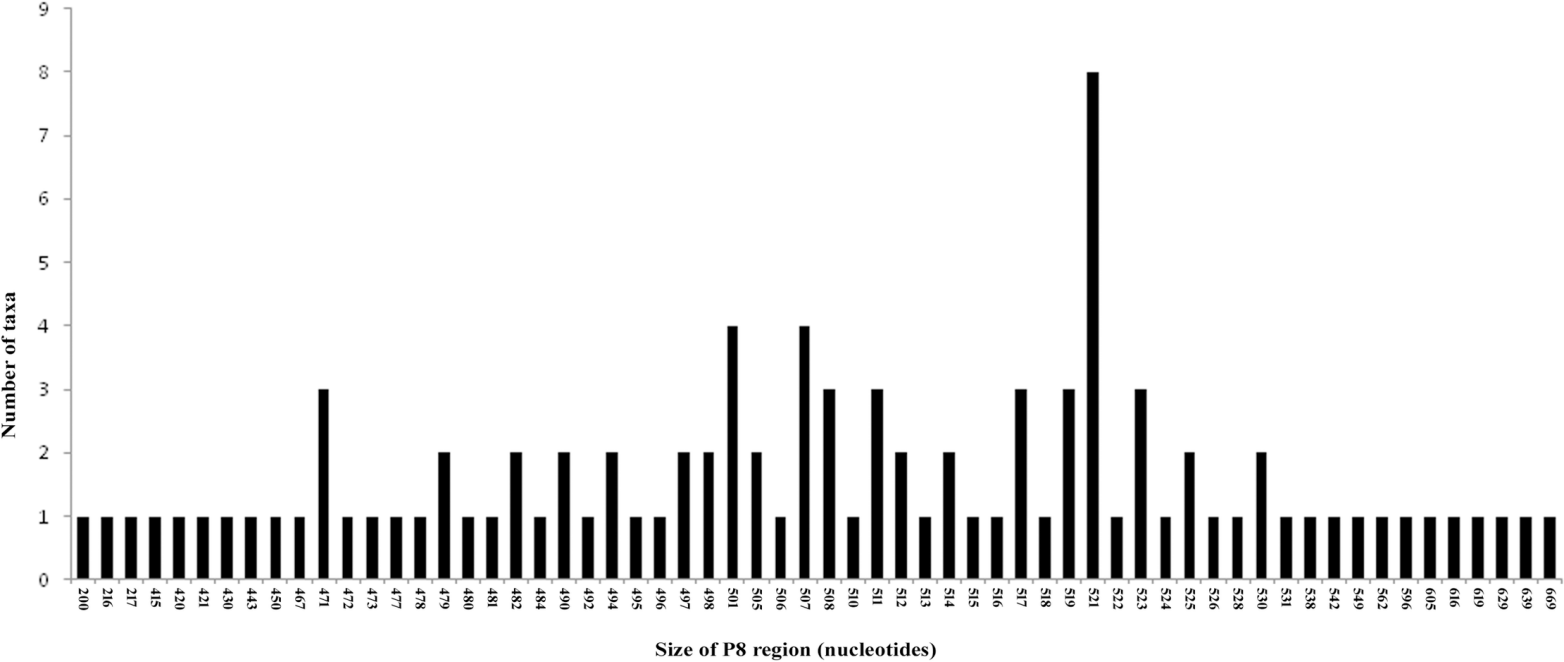
Length variation of the *trn*L(UAA) intron P8 regions of the 98 taxa of orchids versus number of taxa sharing common lengths.

The downloaded *trn*L sequences for the 125 accessions contained many with incomplete sequences. After establishing the borders, only 98 taxa with complete sequence of the P8 region could be selected for the analysis. The length of the P8 regions varied from 200 nucleotides (*Aerides rosea*) to 669 nucleotides (*Dendrophylax sallei*) (Fig 1, Table 1). Forty two taxa had unique lengths, while as many as eight shared a common length of 521 nucleotides (*Aeranthes arachnites*, *Aeranthes grandiflora*, *Angraecum conchiferum*, *Angraecum dives*, *Angraecum leonis*, *Bonniera appendiculata*, *Phalaenopsis amboinensis*, and *Phalaenopsis modesta*). Within the genus *Phalaenopsis*, which is represented by 23 species in the present study, length variation of the P8 regions ranged from 494 (*P*. *tetraspis*) to 538 nucleotides (*P*. *lindenii*); while in eight taxa of *Vanda*, it ranged from 471 (*V*. *testaceae*) to 562 nucleotides (*V*. *grifithii*).

**Table 1:** Sequence lengths of *trn*L P8 regions of 98 orchid taxa along with the restriction sites for the endonucleases *Ags*I, *Apo*I, *Tsp*DTI and *Vsp*I.

Taxa	GenBank Accession No.	Length of P8 region (nucleotides)	Restriction endonucleases and their respective restriction sites (nucleotides)			
			*Ags*1 (TTSAA)	*Apo*1 (RAATTY)	*Tsp*DTI (ATGAA)	*Vsp*I (ATTAAT)
*Acampe ochracea*	DQ091438	421	259, 265, 293, 357, 400, 406	146, 180, 344, 380	27, 193, 289, 345, 361	121, 192
*Acampe papillosa*	DQ091439	420	259, 265, 293, 357, 400	146, 180, 344, 380	27, 193, 289, 345, 361, 418	121, 192
*Aeranthes arachnites*	DQ091536	521	259, 265, 293, 357, 400	146, 180, 344, 380	27, 193, 289, 345, 361, 418	121, 192
*Aeranthes grandiflora*	DQ091537	521	238, 400, 428, 434, 452, 500	145, 238, 445	27, 263, 405, 463, 519	120, 301
*Aerides crassifolia*	EF670402	492	195, 397, 403, 428, 471	146, 189, 196, 415, 451, 472	27, 201, 221, 226, 318, 374, 432	121, 263
*Aerides crispa*	EF670407	484	189, 361, 389, 395, 412, 420, 463	140, 183, 190, 407, 443, 464	27, 195, 215, 310, 366, 424	115, 230, 241
*Aerides krabiensis*	EF670404	217	196	155, 190, 197	27, 202	130
*Aerides multiflora*	DQ194983	216	195	146, 189, 196	27, 190, 201	121
*Aerides odorata*	EF670389	450	166, 327, 355, 361, 386, 429	125, 160, 167, 373, 409, 430	27, 172, 192, 197, 276, 332, 390	100, 221
*Aerides rosea*	EF670405	200	138, 179	138, 173, 180	27, 174, 185	113
*Aerides sukauensis*	EF670408	479	195, 356, 384, 390, 415, 458	189, 196, 402, 459	27, 201, 221, 305, 361, 419	121, 236
*Ancistrorhynchus cephalotes*	EF670435	619	345, 526, 532, 598	145, 345, 544	27, 199, 370, 503	120, 603, 120, 603
*Angraecum calceolus*	DQ091546	505	238, 420, 484	145, 238, 431	27, 263, 447	120, 294, 489
*Angraecum conchiferum*	DQ091539	521	238, 428, 434, 500	145, 238, 446	27, 263, 405, 463	120, 301, 505
*Angraecum dives*	DQ091547	521	238, 400, 428, 434, 500	145, 238, 446	27, 263, 405	
*Angraecum eichlerianum*	AF506341	639	145, 238, 446	147, 359, 558	27, 194, 201, 384, 517, 575	122, 617, 623
*Angraecum florulentum*	DQ091550	513	237, 392, 420, 426, 492	145, 237, 438	27, 262, 397, 455	120, 293, 497
*Angraecum leonis*	DQ091551	521	238, 400, 428, 434, 500	145, 238, 446	27, 263, 405, 463	120, 301, 505
*Angraecum magdalenae*	AF519973	616	334, 490, 524, 595	145, 254, 334, 536	27, 210, 359, 495, 553	120, 243, 391, 600
*Angraecum rutenbergianum*	DQ091548	443	321, 349, 355, 421	145, 367	27, 326, 384	120, 222, 426
*Angraecum teres*	DQ091552	542	145, 252, 266, 421, 449, 455, 521	266, 467	27, 291, 426, 484	120, 322, 526
*Arachnis labrosa*	GU185926	478	195, 357, 385, 391, 416, 459	146, 189, 196, 403, 460	27, 201, 221, 306, 362, 420	121, 236, 250
*Ascocentrum curvifolium*	EF670423	494	187, 371, 399, 405, 430, 473, 479	146, 180, 188, 417, 438, 453	27, 193, 213, 320, 376, 434	121, 228, 250, 265
*Beclardia macrostachya*	DQ091497	523	259, 411, 439, 445, 502	145, 259, 457	27, 192, 198, 284, 416	120, 312, 507
*Bonniera appendiculata*	DQ091541	521	238, 428, 434, 451, 500	145, 238, 446	27, 263, 405, 463	120, 301, 505
*Christensonia vietnamica*	EF670413	497	200, 367, 395, 401, 426, 476, 482	146, 193, 201, 413, 434, 449	27, 206, 226, 316, 372, 43027, 206, 226, 316, 372, 430	121, 247
*Cleisostoma racemiferum*	GU185928	525	231, 402, 430, 436, 461, 504, 510	146, 224, 232, 448, 484	27, 163, 214, 230, 237, 257, 351, 407, 465	121, 204, 273, 296
*Cryptopus elatus*	DQ091450	523	240, 402, 430, 436, 453, 502	145, 240, 448	27, 265, 407, 465	120, 303, 507
*Cryptopus paniculatus*	DQ091451	514	233, 393, 421, 427, 444, 493	145, 233, 439	27, 258, 398, 456	120, 498
*Dendrophylax sallei*	AY147234	669	186, 379, 509, 582, 648	145, 379, 594	27, 192, 210, 272, 404, 553, 560, 611	120, 653
*Dimorphorchis rossii var*. *graciliscapa*	EF670429	519	214, 396, 424, 430, 455, 498, 504	141, 208, 215, 278, 284, 442, 478	27, 220, 240, 345, 401, 459	116, 255, 269
*Holcoglossum kimballianum*	EF670419	629	218, 506, 534, 540, 565, 608	146, 212, 219, 552, 588, 609	27, 224, 244, 455, 511, 569	121, 255, 262, 285, 292, 315, 322
*Holcoglossum subulifolium*	EF670409	467	187, 344, 372, 378, 446	146, 181, 188, 390, 426, 447	27, 193, 213, 293, 349, 407	121, 224
*Jumellea maxillarioides*	DQ091554	506	238, 408, 419, 485	145, 238, 431	27, 263, 392, 450	120, 286
*Jumellea sagittata*	DQ091555	508	238, 387, 415, 421, 487	145, 238, 433	27, 263, 390, 448	120, 288, 492
*Lemurorchis madagascariensis*	DQ091556	528	238, 407, 435, 441, 507	145, 238, 453	27, 412, 470	120, 512
*Listrostachys pertusa*	DQ091509	524	238, 403, 431, 437, 454, 503	145, 238, 398, 449	27, 157, 263, 408, 466	120
*Luisia trichorrhiza*	EF670428	605	213, 482, 510, 516, 541, 584	146, 207, 214, 376, 499, 528, 564, 585	219, 239, 243, 259, 263, 279, 387, 431, 487, 545	121, 303
*Luisia tristis*	EF670426	596	219, 473, 501, 507, 532, 575	161, 213, 220, 490, 519, 576	225, 245, 249, 265, 269, 285, 374, 393, 422, 478, 536	136, 309
*Microcoelia stolzii*	DQ091530	480	221, 359, 387, 393, 459	145, 221, 354, 405	27, 309, 364, 422	120
*Neofinetia falcata*	EF670421	497	187, 374, 402, 408, 433, 476, 482	146, 181, 188, 391, 420, 456	27, 193, 213, 273, 323, 379, 437	121, 228, 237, 253
*Oeonia rosea*	DQ091452	516	395, 423, 429, 495	145, 233, 441	27, 258, 400, 458	120, 296, 500
*Paraphalaenopsis labukensis*	EF670425	507	384, 412, 418, 443	146, 401, 430, 466, 487	27, 210, 217, 222, 229, 314, 333, 389, 447	121, 250, 264
*Pelatantheria insectifera*	GU185931	482	187, 383, 389, 406, 414, 461, 467	146, 180, 188, 401, 422, 437	27, 193, 213, 320, 382, 388, 418	121, 260
*Phalaenopsis amboinensis*	AY273643	521	178, 398, 426, 432, 457, 500, 506	137, 172, 179, 444, 480	27, 184, 204, 211, 403, 461	112, 226, 234, 286
*Phalaenopsis bellina*	AY273632	495	178, 372, 400, 406, 431, 474, 480	137, 172, 179, 418, 454	27, 184, 204, 377, 435	112, 219, 227, 246, 260
*Phalaenopsis celebensis*	AY265799	507	187, 384, 412, 418, 443, 486, 492	146, 181, 188, 430	27, 193, 213, 389, 447	121, 228, 242, 262
*Phalaenopsis corningiana*	AY273670	508	178, 385, 413, 419, 444, 487, 493	137, 172, 179, 431, 467	27, 184, 204, 390, 448	112, 219, 235, 249
*Phalaenopsis cornu-cervi*	AY265751	501	378, 406, 412, 437, 480, 486	137, 172, 424	27, 184, 204, 383, 441	112, 219, 227, 242, 256
*Phalaenopsis deliciosa*	DQ091444	415	292, 320, 326, 351, 394, 400	146, 181, 338, 374	27, 193, 297, 355	121
*Phalaenopsis inscriptiosinensis*	AY273673	498	178, 375, 403, 409, 434, 477, 483, 676, 873, 901, 907, 932, 975, 981	137, 172, 179, 421, 457, 635, 670, 677, 919, 955	27, 184, 204, 380, 438, 525, 682, 702, 878, 936	112, 219, 241, 263, 610, 717, 739, 761
*Phalaenopsis lamelligera*	AY273679	501	178, 378, 406, 412, 437, 480, 486	137, 172, 179, 424, 460	27, 184, 204, 321, 383, 441	112, 219, 227, 242, 256
*Phalaenopsis lindenii*	AY273649	538	211, 415, 443, 449, 474, 517, 523	146, 205, 212, 461, 497	27, 217, 237, 420, 478	121, 252, 270, 284
*Phalaenopsis lueddemanniana*	AY273640	517	394, 422, 428, 453, 496, 502	137, 172, 179, 440, 476	27, 184, 204, 399, 457	112, 219, 227, 246, 268
*Phalaenopsis maculata*	AY273641	519	396, 424, 430, 455, 498, 504	147, 189, 197, 442, 478	27, 201, 222, 401, 459	122, 254, 268
*Phalaenopsis mannii*	AF519969	523	185, 400, 428, 434, 459, 502, 508	137, 179, 186, 446, 482	27, 191, 211, 405, 463	112, 226, 234, 267, 288
*Phalaenopsis mariae*	AY265770	519	396, 424, 430, 455, 498, 504	137, 172, 179, 442, 478	27, 184, 204, 231, 401, 459	112, 219, 227, 247, 261, 284
*Phalaenopsis micholitzii*	AY273701	505	178, 382, 410, 416, 441, 484, 490	137, 172, 179, 428, 464	27, 184, 204, 387, 445	112, 219, 227, 246, 260
*Phalaenopsis modesta*	AY265793	521	178, 398, 426, 432, 457, 500, 506	137, 172, 179, 444, 480	27, 184, 204, 403, 461	112, 236, 262, 272, 286
*Phalaenopsis pantherina*	AY273666	501	178, 378, 406, 412, 437, 480, 486	137, 172, 179, 424, 460	27, 184, 204, 217, 321, 383, 441	112, 219, 242, 256
*Phalaenopsis philippinensis*	AY273656	511	192, 388, 416, 422, 447, 490, 496	151, 186, 193, 434, 470	27, 198, 218, 393, 451	126, 245, 259
*Phalaenopsis pulchra*	AY273639	517	394, 422, 428, 453, 496, 502	137, 179, 440, 476	27, 204, 399, 457	112, 218, 226, 245, 267, 281
*Phalaenopsis schilleriana*	AY265781	511	192, 388, 416, 422, 447, 490, 496	151, 186, 193, 434, 470	27, 198, 218, 393, 451	126, 245, 259
*Phalaenopsis stuartiana*	AY273654	507	384, 412, 418, 443, 486, 492	146, 181, 430, 466	27, 193, 213, 389, 447	121, 240, 254
*Phalaenopsis sumatrana*	AY273695	508	178, 385, 413, 419, 444, 487, 493	137, 172, 179, 431, 467	27, 184, 204, 390, 448	112, 219, 235, 249
*Phalaenopsis tetraspis*	AY265784	494	178, 371, 399, 405, 430, 473, 479	137, 172, 179, 417, 453	27, 184, 204, 376, 434	112, 219, 235, 249, 259
*Phalaenopsis violacea*	AY273635	507	178, 384, 412, 418, 443, 486, 492	137, 172, 179, 430, 466	27, 184, 204, 389, 447	112, 219, 227, 246, 272
*Pomatocalpa diffusum*	EF670432	471	187, 348, 376, 382, 399, 407, 450, 456	146, 180, 188, 394	213, 297, 353, 411	121, 228
*Pomatocalpa spicatum*	EF670431	490	146, 187, 367, 395, 418, 426, 469	146, 180, 188, 413, 470	27, 193, 213, 316, 372, 430	121, 228, 247
*Rangaeris amaniensis*	DQ091512	517	238, 424, 430, 496	145, 238, 442	27, 263, 401, 408, 459	120
*Rangaeris rhipsalisocia*	DQ091511	515	145, 239, 427, 433, 494	239, 440	27, 185, 264, 404, 457	120
*Renanthera imschootiana*	GU185932	472	147, 188, 349, 377, 383, 400, 408, 451, 457	147, 182, 189, 343, 395, 416, 431	27, 194, 214, 298, 354, 412	122, 229
*Renanthera matutina*	AY273688	498	146, 375, 409, 434, 483	146, 181, 188, 421	27, 213, 324, 380	121, 228, 246, 256, 275, 448
*Renantherella histrionica*	AY273692	496	146, 187, 386, 414, 420, 445	146, 181, 188, 380, 432, 468	27, 193, 208, 213, 335, 391, 449	121, 228, 241, 265
*Rhipidoglossum kamerunense*	DQ091492	512	238, 418, 424, 491	145, 238, 437	27, 263, 269, 395, 454	120, 496
*Rhipidoglossum rutilum*	DQ091494	522	238, 428, 434, 501	145, 238, 447	27, 263, 269, 405, 464	120, 506
*Rhipidoglossum subsimplex*	DQ091496	512	238, 418, 424	145, 238, 437	27, 263, 269, 395, 454	120, 496
*Rhynchostylis gigantea*	EF670411	479	195, 356, 384, 390, 407, 415, 458	146, 189, 196, 402, 438, 459	27, 73, 201, 221, 305, 361, 419	121, 236, 250
*Rhynchostylis retusa*	EF670424	501	378, 406, 412, 429, 480	146, 424, 445, 460, 481	27, 201, 327, 383, 441	121, 226, 246, 265
*Sedirea japonica*	EF670433	473	350, 378, 384, 401, 409, 452	146, 396, 432, 453	27, 193, 200, 355, 413	121, 222, 244
*Seidenfadenia mitrata*	EF670414	490	200, 367, 395, 401, 426, 469, 475	146, 193, 201, 413, 449	27, 206, 226, 316, 372, 430	121, 247
*Smitinandia micrantha*	EF670415	481	187, 319, 325, 353, 410, 417, 460, 466	146, 180, 188, 404, 440	27, 193, 213, 349, 405, 421	121, 228, 252
*Sobennikoffia humbertiana*	DQ091558	514	238, 393, 421, 427, 493	145, 238, 439	27, 263, 398, 456	120, 295, 498
*Trichoglottis atropurpurea*	DQ091440	430	330, 335, 341, 366, 409	146, 188, 302, 353, 410	27, 201, 256, 312, 370	121, 196, 200
*Trichoglottis bipunctata*	EF670430	531	187, 436, 442, 459, 467, 510	146, 180, 188, 403, 454, 511	27, 193, 219, 356, 412, 471	121, 248, 279
*Tridactyle crassifolia*	DQ091515	530	55, 145, 244, 437, 443, 509	244, 455	27, 269, 414, 472	120
*Tridactyle filifolia*	DQ091516	530	145, 244, 437, 443, 509	244, 455	27, 269, 414, 472	120
*Tridactyle tanneri*	DQ091520	518	239, 358, 425, 431, 497	146, 239, 443	27, 158, 264, 402, 460	121
*Vanda alpina*	GU185933	526	187, 403, 431, 437, 462, 505, 511	146, 180, 188, 449, 485	27, 193, 213, 352, 408, 466	121, 228, 259, 277, 295
*Vanda coerulea*	GU185935	549	208, 426, 454, 460, 485, 528, 534	146, 201, 209, 472, 508	27, 214, 234, 375, 431, 489	121, 257, 271, 275, 294, 311, 328
*Vanda flabellata*	EF670410	477	187, 354, 382, 388, 413, 456, 462	146, 180, 187, 400, 436	27, 193, 213, 303, 359, 417	121, 234
*Vanda griffithii*	GU185934	562	187, 444, 472, 478, 541, 547	146, 180, 188	27, 193, 213, 375, 393, 449, 503	121, 228, 245, 276, 302, 320, 338
*Vanda luzonica*	AY273699	511	187, 387, 415, 421, 447, 490, 496	146, 180, 188, 434, 470	27, 193, 213, 336, 392, 451	121, 262
Vanda motesiana*	GU185938	510	187, 387, 415, 421, 438, 446, 489, 495	180, 188, 433, 469	27, 193, 213, 336, 392, 450	121, 228, 248, 262
*Vanda stangeana*^*#*^	GU185939	525	187, 402, 430, 436, 461, 504, 510	146, 180, 188, 448, 484	27, 193, 220, 230, 332, 350, 406, 465	121, 245, 277
*Vanda testacea*	GU185940	471	187, 348, 376, 382, 399, 407, 450, 456	180, 188, 394, 430	27, 193, 213, 297, 353, 411	121, 228, 242
*Vandopsis gigantea*	EF670417	482	187, 348, 376, 382, 407	146, 180, 188, 394, 430, 451, 462	27, 193, 213, 297, 353, 411	121, 228, 242
*Vandopsis lissochiloides*	EF670418	471	187, 348, 376, 382, 407, 450	146, 180, 188, 394, 451	27, 193, 213, 297, 353, 411	121, 228, 242

R = A or G, S = C or G, Y = C or T; while submitting to GenBank the *trn*L sequences

Vanda motesiana* was entered as *V*. *stangeana* and

*V*. *stangeana*^*#*^ entered as *V*. *tessellate*

### In silico analysis of restriction site polymorphism

**Fig 2:**
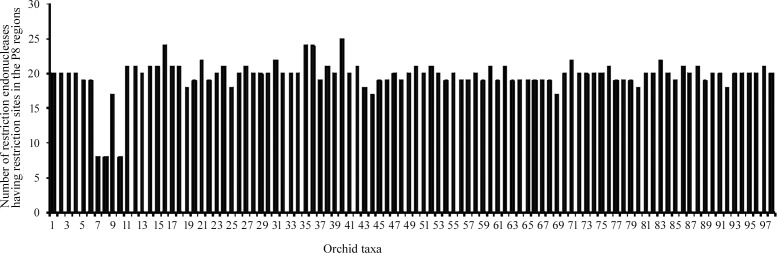
Graph representing diversity of restriction endonucleases showing recognition sites in the P8 regions of the *trn*L(UAA) intron of the 98 taxa of orchids (1–98 represent names of 98 orchids in alphabetical order as presented in Table 1).

The result of the in silico restriction site analysis is presented in Table 1. Of all the commercially available restriction endonucleases being tried for the in silico restriction mapping using the online software ‘RestrictionMapper’, 35 endonucleases (*Afl*II, *Acc*I, *Ags*I, *Apo*I, *Asu*II, *Bci*VI, *Bcl*I, *Bda*I, *Bgl*II, *Bsa*AI, *Bsa*BI, *Bsm*I, *Csp*CI, *Eco*57I, *Eco*57MI, *Eco*RI, *Eco*RV, *Hae*IV, *Mfe*I, *Mbo*II, *Msl*I, *Nde*I, *Psi*I, *Sml*I, *Sna*BI, *Spe*1, *Ssp*I, *Tfi*I, *Tsp*DTI, *Tsp*GWI, *Tsp*RI, *Vsp*I, *Xba*I, *Xho*II and *Xmn*I) were found to have recognition sites in the P8 region. Number of restriction endonucleases digesting the region varied from eight in *Aerides krabiensis*, *A*. *odorata* and *A*. *rosea* to 25 in *Microcoelia stolzii* (Fig 2). Again, from amongst the 35 restriction endonucleases, only three, viz., *Ags*I, *Apo*I and *Tsp*DTI turned out to have recognition sites across all the 98 taxa being studied; while *Vsp*I had in all excepting *Angraecum dives*. Hence, the above four were selected for subsequent analysis of the restriction site polymorphism.

### Species discrimination by single restriction endonuclease

**Fig 3:**
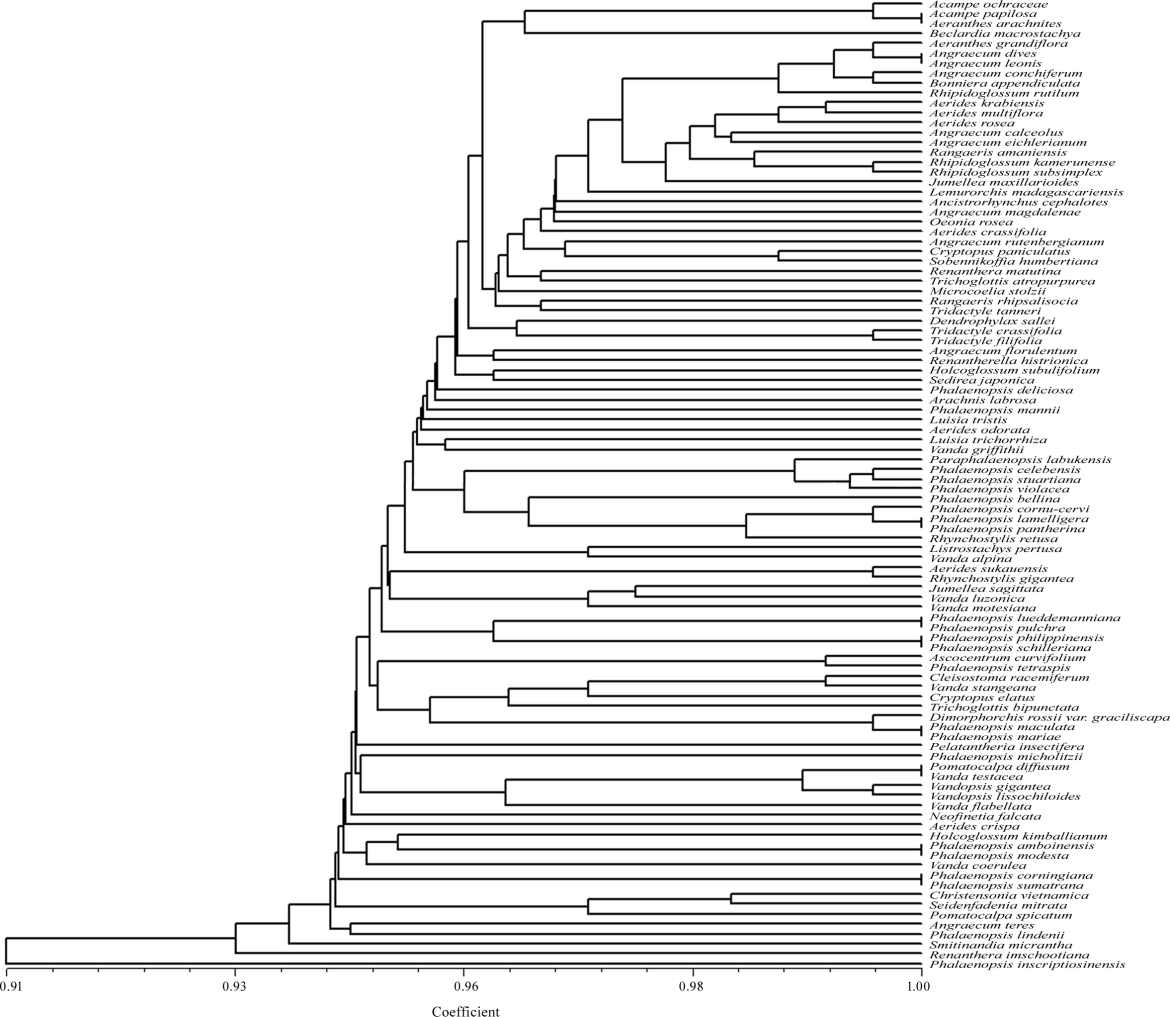
UPGMA dendrogram based on SAHN clustering of data from in silico restriction site polymorphism analysis of the *trn*L(UAA) intron P8 region using the restriction endonuclease *Ags*I among the 98 taxa of orchids.

The restriction sites recognized by *Ags*I ranged from one (*Aerides krabiensis* and *A*. *multiflora*) to 14 (*Phalaenopsis inscriptiosinensis*) (Table 1). From the dendrogram constructed by the UPGMA method which showed similarity coefficient ranging from 0.91 to 1.00 (Fig 3), it was found that *Ags*I exhibited species specific restriction sites in 80 taxa; however, it could not provide discrimination for 18 taxa which included the taxon pairs *Acampe papillosa* and *Aeranthes arachnites; Angraecum dives* and *Angraecum leonis*; *Phalaenopsis lamelligera* and *P*. *pantherina*, *P*. *lueddemanniana* and *P*. *pulchra*; *Phalaenopsis philippinensis* and *P*. *schilleriana*; *Phalaenopsis maculata* and *P*. *mariae*; *Phalaenopsis amboinensis* and *P*. *modesta*; *Phalaenopsis corningiana* and *P*. *sumatrana; Pomatocalpa diffusum* and *Vanda testacea*. Each of these taxon pairs shared the same *Ags*I restriction sites.

**Fig 4:**
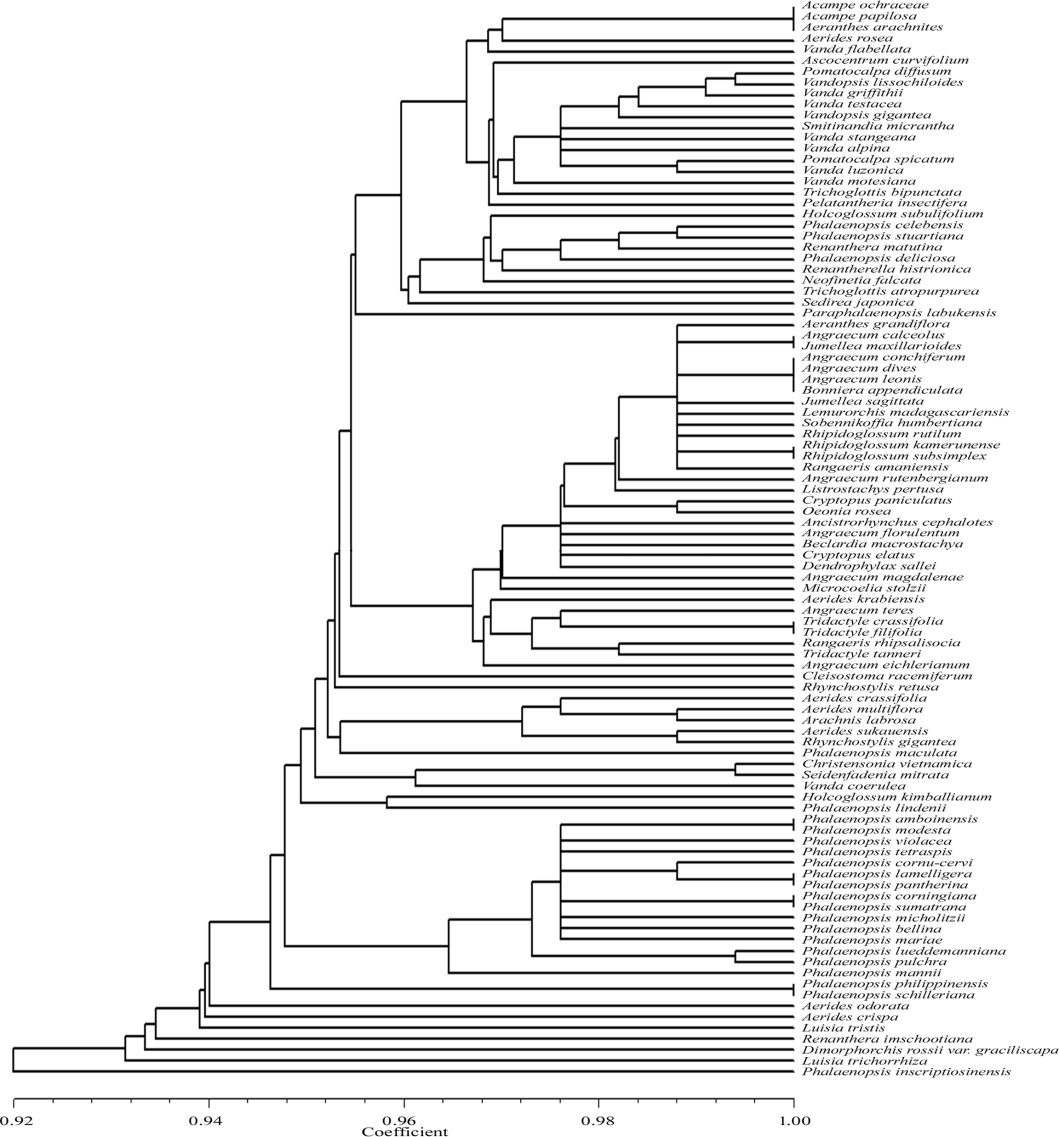
UPGMA dendrogram based on SAHN clustering of data from in silico restriction site polymorphism analysis of the *trn*L(UAA) intron P8 region using the restriction endonuclease *Apo*I among the 98 taxa of orchids.

The *Apo*I recognized restriction sites ranged from two (in six taxa) to ten (*Phalaenopsis inscriptiosinensis*) (Table 1). As evidenced from the UPGMA dendrogram (Fig 4), of the 98 taxa analysed *Apo*I digestion discriminated 77 species, however; it could not show species specific restriction sites in 21 taxa. The following taxon pairs or groups shared the same restriction sites, *Acampe ochraceae*, *Acampe papillosa* and *Aeranthes arachnites*, *Angraecum calceolus* and *Jumellea maxillarioides*, *Angraecum conchiferum*, *Angraecum dives*, *Angraecum leonis* and *Bonniera appendiculata*, *Rhipidoglossum kamerunense* and *Rhipidoglossum subsimplex*, *Tridactyle crassifolia* and *Tridactyle filifolia*, *Phalaenopsis amboinensis* and *P*. *modesta*, *Phalaenopsis lamelligera* and *P*. *pantherina*, *Phalaenopsis corningiana* and *P*. *sumatrana*, *Phalaenopsis philippinensis* and *P*. *schilleriana*. This dendrogram showed similarity coefficient ranging from 0.92 to 1.00.

**Fig 5:**
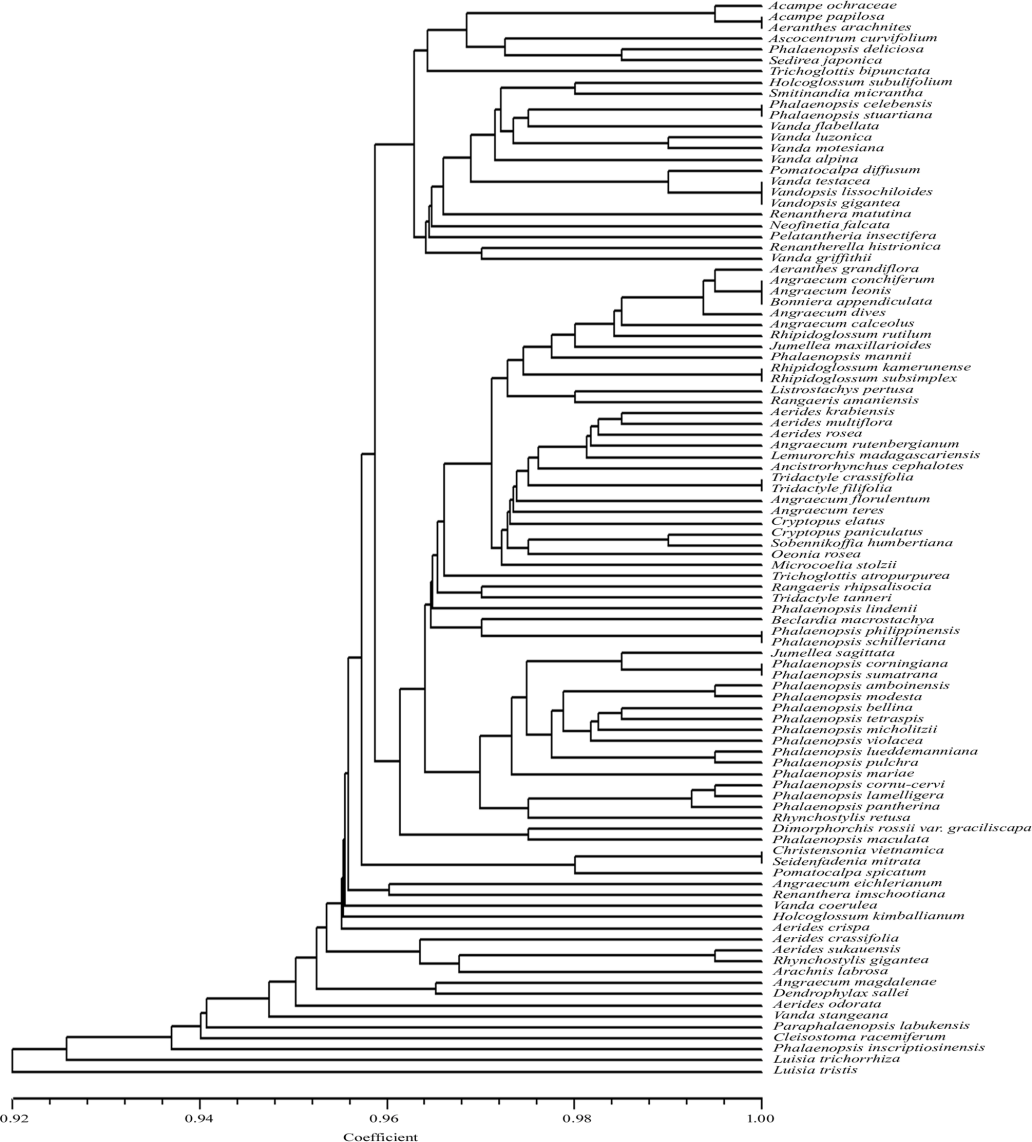
UPGMA dendrogram based on SAHN clustering of data from in silico restriction site polymorphism analysis of the *trn*L(UAA) intron P8 region using the restriction endonuclease *Tsp*DTI among the 98 taxa of orchids.

*Tsp*DTI exhibited species specific restriction patterns for 78 taxa and could not discriminate 20 taxa (Table 1, Fig 5). These twenty included the taxon pairs or groups *Acampe papillosa* and *Aeranthes arachnites; Christensonia vietnamica* and *Seidenfadenia mitrata*; *Phalaenopsis celebensis* and *Phalaenopsis stuartiana*; *Phalaenopsis philippinensis* and *P*. *schilleriana; Phalaenopsis corningiana* and *P*. *sumatrana; Vanda testacea*, *Vandopsis lissochiloides* and *Vandopsis gigantean*; *Rhipidoglossum kamerunense* and *Rhipidoglossum subsimplex; Tridactyle crassifolia* and *Tridactyle filifolia*. The UPGMA dendrogram showed similarity coefficient ranging from 0.92 to 1.00.

**Fig 6:**
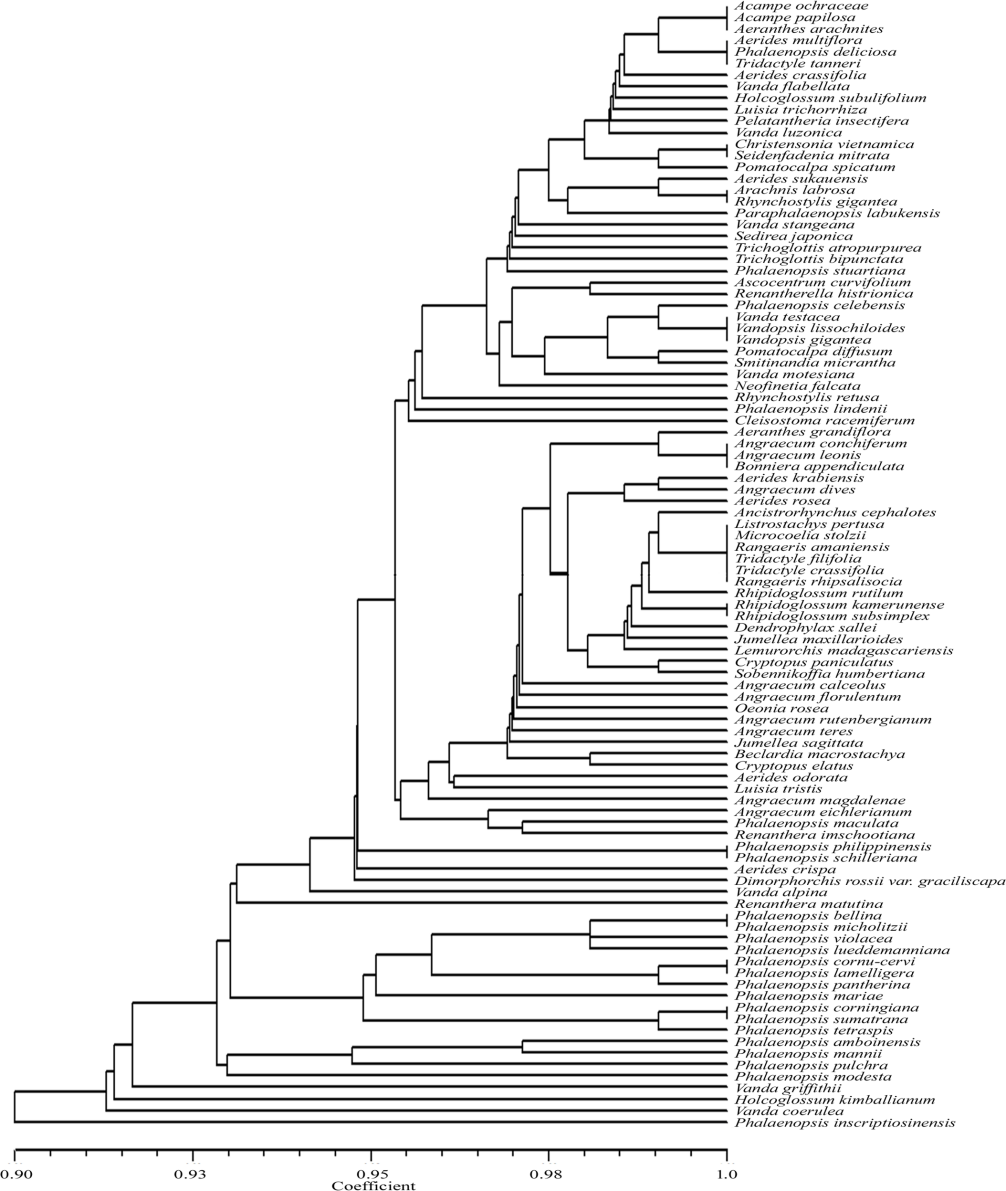
UPGMA dendrogram based on SAHN clustering of data from the in silico restriction site polymorphism analysis of the *trn*L(UAA) intron P8 region using the restriction endonuclease *Vsp*I among the 98 taxa of orchids.

*Vsp*I also exhibited species specific restriction patterns for 66 taxa and could not discriminate 32 taxa (Table 1, Fig 6). These 32 include the taxon pairs or groups, *Acampe ochraceae*, *Acampe papillosa* and *Aeranthes arachnites; Aerides multiflora*, *Phalaenopsis deliciosa* and *Tridactyle tanneri*; *Christensonia vietnamica* and *Seidenfadenia mitrata*; *Arachnis labrosa* and *Rhynchostylis gigantean*; *Vanda testacea*, *Vandopsis lissochiloides* and *Vandopsis gigantea*; *Angraecum conchiferum*, *Angraecum leonis* and *Bonniera appendiculat; Listrostachys pertusa*, *Microcoelia stolzii*, *Rangaeris amanuensis*, *Rangaeris rhipsalisocia*, *Tridactyle crassifolia* and *Tridactyle filifolia; Rhipidoglossum kamerunense* and *Rhipidoglossum subsimplex; Phalaenopsis philippinensis* and *P*. *schillerian; Phalaenopsis bellina* and *Phalaenopsis micholitzii*; *Phalaenopsis cornu-cervi* and *Phalaenopsis lamelligera*; *Phalaenopsis corningiana* and *P*. *sumatrana*. The UPGMA dendrogram showed similarity coefficient ranging from 0.90 to 1.00.

### Species discrimination using combined data of restriction endonucleases

**Fig 7:**
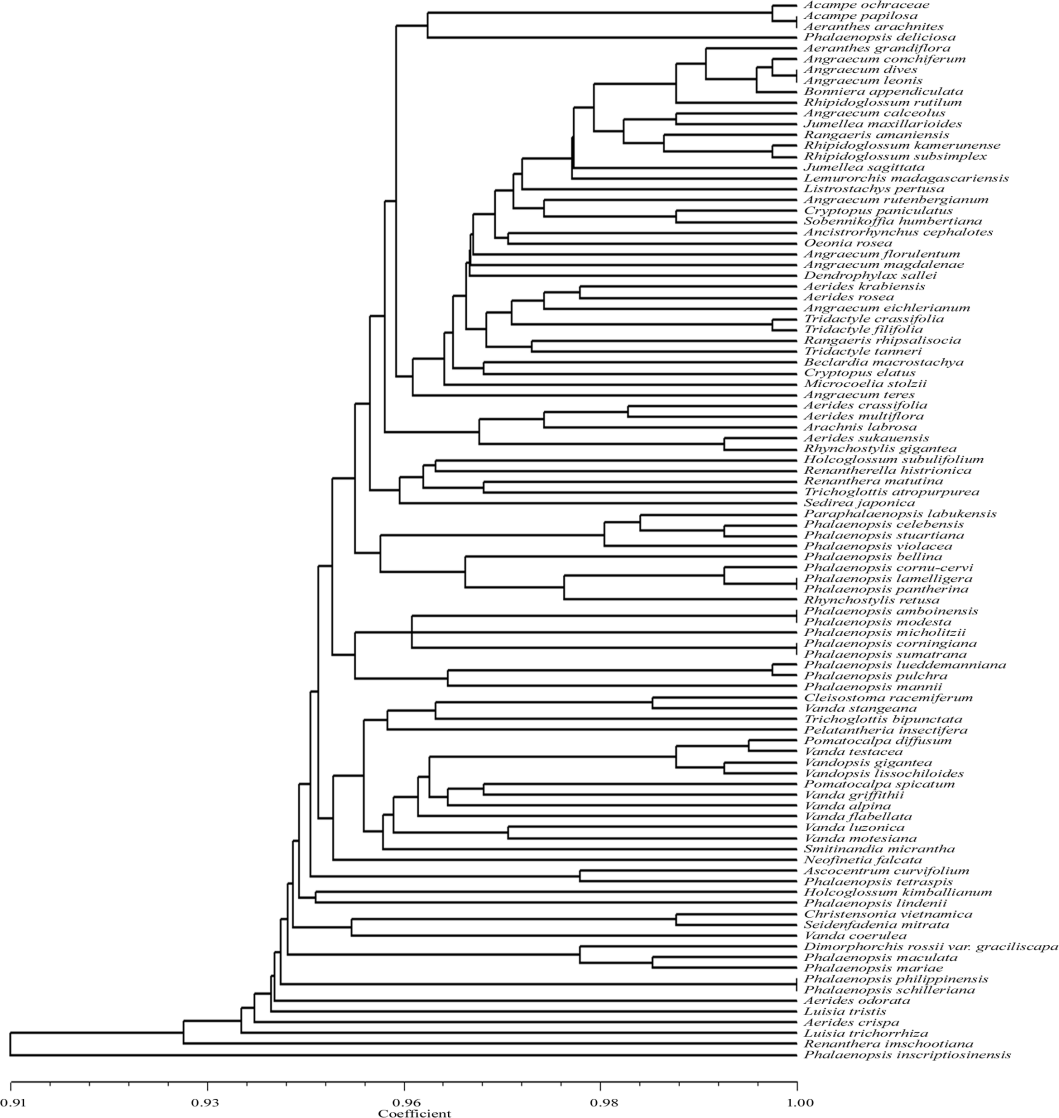
UPGMA dendrogram based on SAHN clustering of the combined data from in silico restriction site polymorphism analyses of the *trn*L(UAA) intron P8 region using the restriction endonuclease *Ags*I and *Apo*I among the 98 taxa of orchids.

Analysis of the in silico restriction mapping using the combined data of *Ags*I and *Apo*I resulted in discrimination of 86 taxa (Fig 7) as against their individual resolutions of 80 and 77 taxa respectively. Those which had exactly the same restriction sites included six taxon pairs, *Acampe papillosa* and *Aeranthes arachnites; Angraecum dives* and *Angraecum leonis*; *Phalaenopsis lamelligera* and *P*. *pantherina; Phalaenopsis amboinensis* and *P*. *modesta*; *Phalaenopsis corningiana* and *P*. *sumatrana; Phalaenopsis philippinensis* and *P*. *schilleriana*. The UPGMA dendrogram showed similarity coefficient ranging from 0.91 to 1.00.

**Fig 8:**
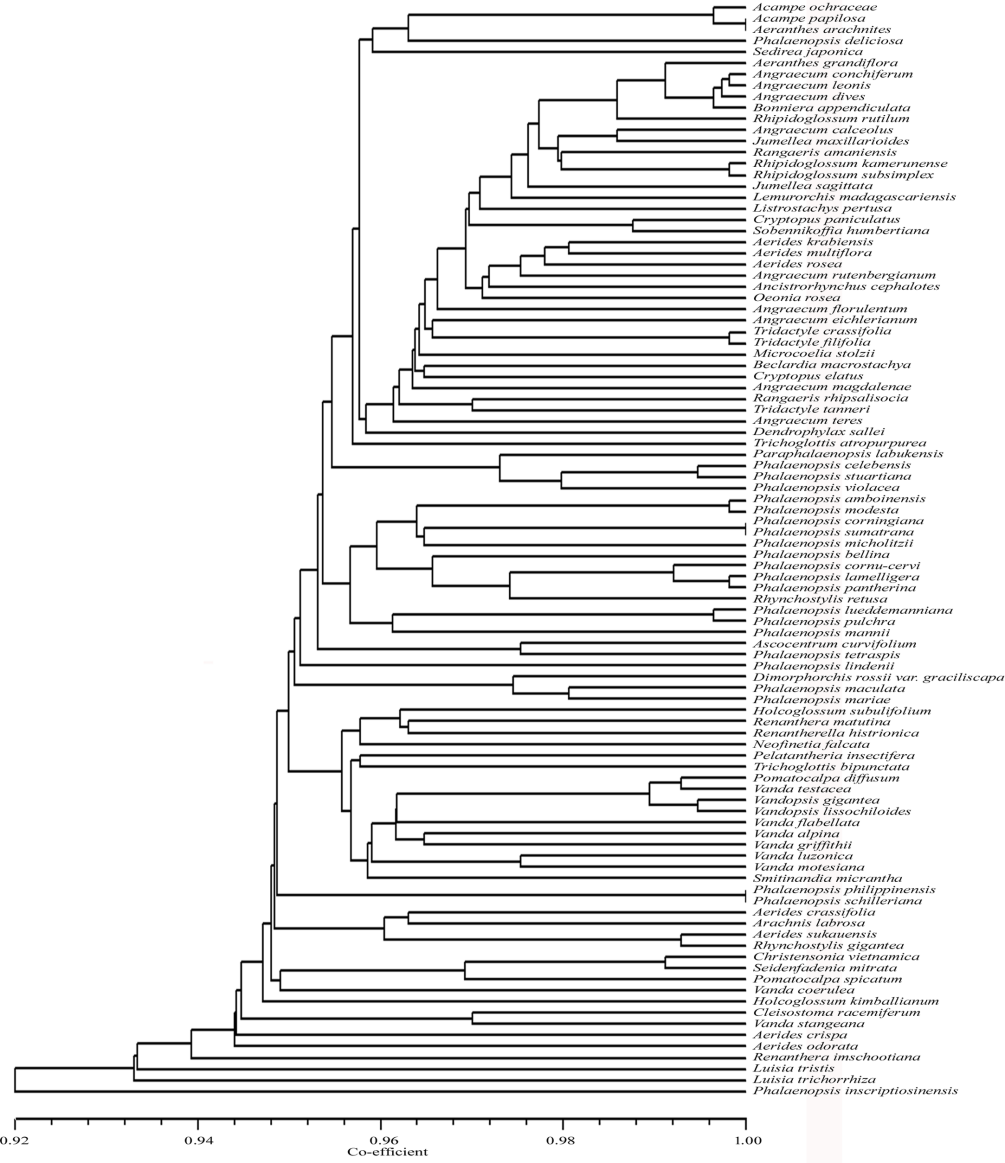
UPGMA dendrogram based on SAHN clustering of the combined data from in silico restriction site polymorphism analyses of the *trn*L(UAA) intron P8 region using the restriction endonuclease *Ags*I, *Apo*I and *Tsp*DTI among the 98 taxa of orchids.

Again, when *Apo*I, *Ags*I and *Tsp*DTI restriction data were combined, 92 taxa could be discriminated while the three taxon pairs which could not be discriminated from each other included *Acampe papillosa* and *Aeranthes arachnites; Phalaenopsis corningiana* and *P*. *sumatrana; Phalaenopsis philippinensis* and *P*. *schilleriana* (Table 1, Fig 8). The UPGMA dendrogram showed similarity coefficient ranging from 0.92 to 1.00.

**Fig 9:**
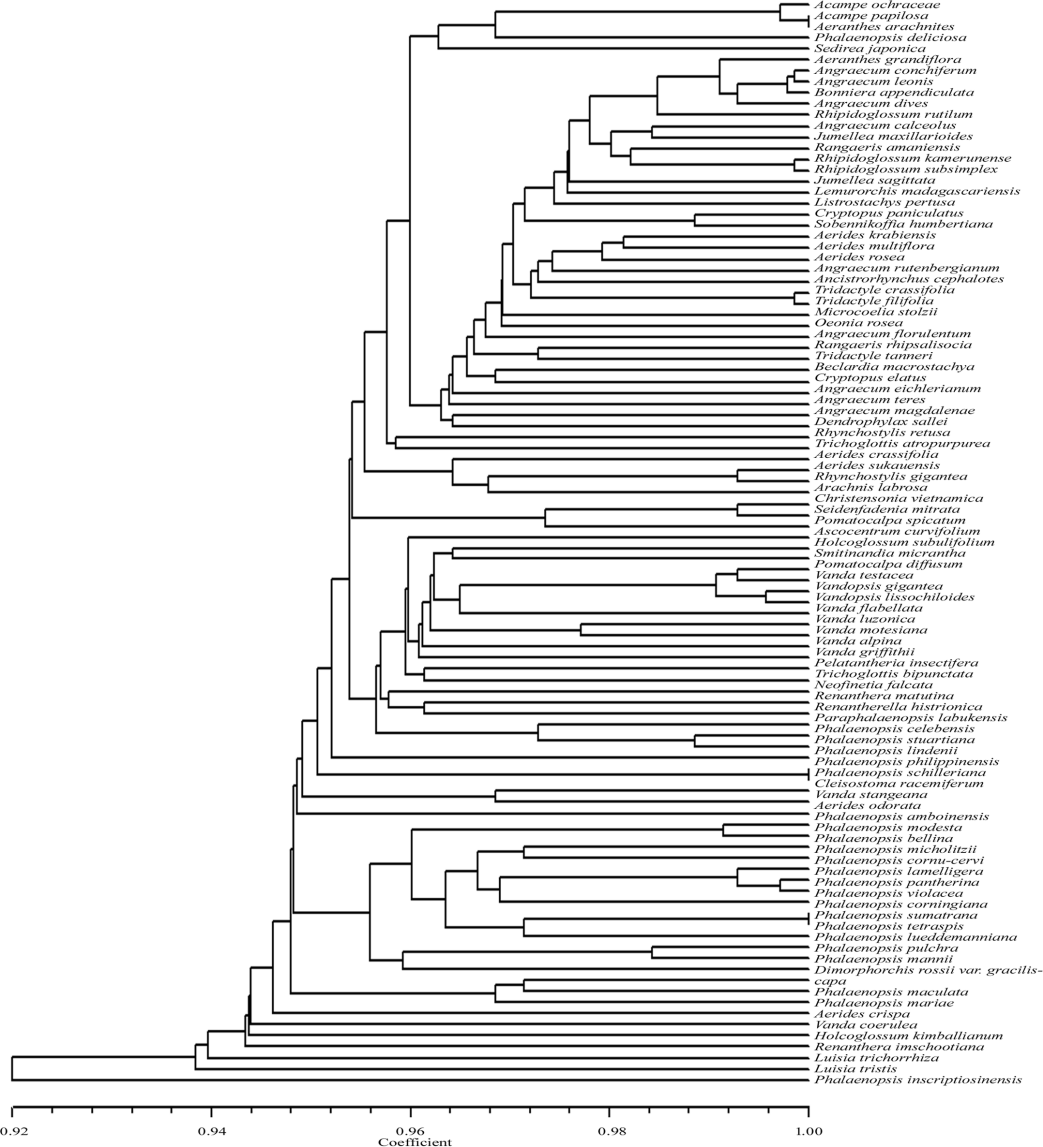
UPGMA dendrogram based on SAHN clustering of the combined data from in silico restriction site polymorphism analyses of the *trn*L(UAA) intron P8 region using the restriction endonuclease *Ags*I, *Apo*I, *Tsp*DTI and *Vsp*I among the 98 taxa of orchids.

Combination of data of all the four enzymes *Apo*I, *Ags*I, *Tsp*DTI and *Vsp*I also gave the same result as that of the above three enzymes with no discrimination of the following three pairs *Acampe papillosa* and *Aeranthes arachnites; Phalaenopsis corningiana* and *P*. *sumatrana; Phalaenopsis philippinensis* and *P*. *schilleriana* (Table 1, Fig 9). The UPGMA dendrogram showed similarity coefficient ranging from 0.92 to 1.00.

## Discussion

The P8 stem-loop region is reported to be the most length-variable region of the *trn*L intron which is due to presence of repeats of various sizes [21]. These repeats together with the enormous length and sequence variation hampered alignment in most of the plant groups being studied for phylogeny or barcoding and therefore, this region is often excluded from analysis of *trn*L intron sequences [14–16]. Due to this exclusion, the results inferred from analysis of the *trn*L intron sequences do not represent the exact information it should naturally infer. By virtue of its hypervariability, the P8 region should be having certain information which may be analysed and interpreted for applying in species level identification or barcoding, if not for phylogenetic inference. As there is difficulty in sequence alignment of the P8 regions, the only other method for analysis should be the restriction analysis. In silico restriction site polymorphism was opted over the conventional PCR-Restriction Fragment Length Polymorphism (PCR-RFLP) as there were already a large number of *trn*L sequences deposited to GenBank. Moreover, the PCR-RFLP may not show the true picture in case two fragments of equal lengths coming from different parts of the sequence cannot be distinguished by observing the gel picture. For plant species identification PCR-RFLP is seldom used, however, a few investigators employed it for identification of mangrove and mangrove associate species [23], fine roots of trees from the Alps [24], *Cinnamomum* spp. [25], upland grassland species from roots [26] and *Vasconcellea* species [27], Dendrobium orchids [28].

### Sequence length analysis

The tribe Vandeae (Family: Orchidaceae) is very robust consisting of 2600 species of monopodial epiphytes under 139 genera. Some previous investigators had already worked on phylogeny of these orchids using *trn*L intron sequences [15–17]. As an objective of the present investigation we aimed to discriminate closely related species belonging to a genus or a subtribe or a tribe. While searching the GenBank database, 125 accessions of *trn*L intron sequence for taxa, under the tribe Vandeae, was available when we started the investigation and they were retrieved for analysis. Upon further investigation 98 out of the 125 sequences had complete P8 hypervariable regions, and thus were considered for the final analysis. Sequence length variation of the P8 regions could discriminate 92 out of the 98 taxa of orchids being investigated. It was observed that the length of the P8 regions of all the taxa being varied from 200 (*Aerides rosea*) to 669 nucleotides (*Dendrophylax sallei*). *Dendrophylax sallei* had 30 more nucleotides longer than *Luisia curtisii* (Orchidaceae) which was reported earlier to be the longest recorded angiosperm P8 (639 nucleotides) [16]. As evidenced from our finding, those taxa sharing a common length did not necessarily belong to the same genus and that there could be great differences in P8 lengths within the same genus, which is in conformity with that of Kocyan et al. [16]. This difference in P8 lengths might be due to slipped-strand mispairing (SSM) resulting into high repetition of A motifs [21, 29].

### Restriction site polymorphism analysis

Our study represents the first ever in silico restriction analysis of the P8 region of *trn*L in an attempt to utilize the genetic information present in it for a meaningful interpretation in species identification of a certain group of angiospermic plants. From all the UPGMA dendrograms generated in this investigation it is evidenced that species belonging to one genus are clustered with those belonging to other genus or genera, or in the other sense they are haphazardly clustered owing to the hypervariable nature of the P8 regions. This showed that the present approach might not be applicable for phylogenetic inference of the taxa being investigated. However, since 95.9% of these taxa could be discriminated based on restriction site polymorphisms and sequence length data, this technique might be adopted for rapid species identification and hence as a plant DNA barcode.

So far there has not been much report on utilization of PCR-RFLP for identification of orchid species except for certain Thai *Dendrobium* orchids using rDNA-ITS and cpDNA regions [28]. Some investigators already showed the efficacy of double digestion or using two or more restriction endonucleases over single in generation of more polymorphic fragments in PCR-RFLP experiments. PCR-RFLP of the chloroplast *trn*S-*psb*C gene regions using a combination of two enzymes, *Hae*III and *Msp*I could successfully identify all the 119 accessions of millet into 7 species [30]. 579 grasses roots were distinguished to ten species using PCR-RFLP of *trn*L intron, with one or two enzyme digest [26]. Again, 16 taxa out of 30 tree species from the Alps were identified using PCR-RFLP with four restriction endonucleases *Taq*I, *Hinf*I, *Rsa*I and *Cfo*I [24]. However, in our investigation, it was observed that as many as 35 restriction endonucleases had their recognition sites in the region. Combined data from analyses of the P8 regions with three restriction endonucleases *Apo*I, *Ags*I and *Tsp*DTI, could discriminate 92 of the 98 taxa based on species specific restriction sites. Hence, the advantage of screening a large number of restriction endonucleases is required for higher success rate in species discrimination of the plant specimens being investigated.

*Aerides rosea*, having the shortest P8 length of 200 nuclotides, showed to have recognition sites of at least eight enzymes, while *Dendrophylax sallei* despite having the longest P8 length (669 nucleotides) had recognition sites for only 20 enzymes. *Microcoelia stolzii* with a P8 length of 480 nucleotides had the maximum number (25) of restriction endonucleases cutting the region. Again, considering the number of recognition sites per restriction endonuclease for an individual taxon, *Phalaenopsis inscriptiosinensis*, with a P8 length of 498 nucleotides, had as many as 14 *Ags*I, 10 *Apo*I, 10 *Tsp*DTI and 8 *Vsp*I recognition sites. Hence, our result showed that the longest P8 length neither had the maximum number of restriction endonuclease recognizing it nor maximum number of recognition sites for an individual enzyme.

It was observed that there were 20 restriction endonucleases having recognition sites in the P8 regions of both *Acampe papillosa* and *Aeranthes arachnites*. These two taxa also exhibited the same number of restriction sites for all the twenty enzymes. The UPGMA trees drawn for each of the four enzymes as well as those for their combined data revealed them to have 100% genetic similarity. Hence, the only information to differentiate them as different species would be their P8 sequence lengths, 420 nucleotides for *Acampe papilosa* and 521 nucleotides for *Aeranthes arachnites*.

*Phalaenopsis corningiana* and *P*. *sumatrana* are treated as two separate species [31–33]. Our investigation revealed that both of them had the same P8 length (508 nucleotides) and identical sequence and hence could not be distinguished from each other as they had similar restriction sites for all the 19 restriction enzymes. Tsai [34] also could not separate the two taxa based on information derived from nrITS, IGS and *atp*B-*rbc*L sequences. The only morphological differences between them are in callus and marking patterns on the petals. Distribution of *P*. *sumatrana* is widespread ranging from Myanmar, Thailand, Vietnam, Indonesia, Malaysia and the Philippines; whereas *P*. *corningiana* is restricted to Borneo and Sarawak. *Phalaenopsis philippinensis* and *P*. *schilleriana* are also endemic to Philippines and they have distinct distinguishing morphological characters. From our analysis, it is observed that both possessed identical P8 sequences and hence identical restriction endonucleases and their restriction sites. Whether these two taxon pairs should be treated as natural hybrids or ecotypes may need evidences from other coding, non-coding sequences or protein markers.

It was suggested that for a gene region to be practical as a DNA barcode the following three criteria must be fulfilled: (*i*) contain significant species-level genetic variability and divergence, (*ii*) possess conserved flanking sites for developing universal PCR primers for wide taxonomic application, and (*iii*) have a short sequence length so as to facilitate current capabilities of DNA extraction and amplification [3]. From our result, it is learnt that P8 regions of the 98 taxa contained good amount of genetic variation either in sequence length or restriction sites of the enzymes being used. Those taxa which could not be discriminated might require understanding of their maternal origin, in case of natural hybridization; plastid DNA barcodes will fail in case of natural hybrids. Second, the primer pair (c and d) used to amplify *trn*L intron is well conserved from brayophytes to angiosperms [35]. The present study also employed this primer pair for both PCR amplification as well as sequencing. Using these primers, the whole *trn*L region could be amplified and sequenced and from it an intact P8 region be retrieved easily, which is an advantage. Third, sequence lengths of the P8 regions observed in the present investigation ranged from 200 to 669 nucleotides which were short enough to be considered as DNA barcodes. The only disadvantage of the P8 region lie in their inability to be aligned due to hypervariability, and hence cannot be used for further processing using standardized phylogenetic or barcoding techniques.

## Conclusions

A technique for molecular identification using sequence length variation and in silico restriction site polymorphism analysis of the *trn*L intron P8 sequence was developed and utilized to discriminate 94 out of 98 taxa of orchids to the level of species. Investigations using this technique for species level discrimination across all the angiospermic families may be tried. The four restriction endonucleases *Apo*I, *Ags*I, *Tsp*DTI and *Vsp*I could be utilized for further analysis of the *trn*L intron P8 sequences of other uninvestigated orchid taxa either in silico or in PCR-RFLP. A plant DNA barcoding system using restriction site polymorphism of the *trn*L P8 region has not been suggested yet; and with this report there is high possibility of using this tool to establish a barcoding system.
